# Human comparative experimental study of surgical treatment of atrial fibrillation by epicardial techniques

**DOI:** 10.1186/1749-8090-8-140

**Published:** 2013-05-31

**Authors:** Jean-Marc El Arid, Thomas Sénage, Claire Toquet, Ousama Al Habash, Antoine Mugniot, Olivier Baron, Jean-Christian Roussel

**Affiliations:** 1 Department of Cardiovascular Surgery, University Hospital of Lille, Lille, France; 2 Department of Thoracic and CardioVascular Surgery, Thorax Institut, University Hospital of Nantes, Nantes, France; 3 Department of Pathology, University Hospital of Nantes, Nantes, France; 4 Hopital Cardiologique, CHRU de Lille, Boulevard du professeur Leclercq, 59037 Lille Cedex, France

**Keywords:** Arrhythmia surgery, Cryoablation, Radiofrequency, Ultrasound, Experimental surgery

## Abstract

**Background:**

To set up an experimental model of cadaveric heart in order to evaluate and compare histologic transmurality of lesions immediately caused by different energy sources of anti-arrhythmic epicardial devices.

**Methods:**

Procedures were performed on a cadaveric human heart in orthotopic position with an ischemic time of 48 h at 37° and supported through the use of cardiopulmonary bypass. Three anti-arrhythmic epicardial devices were studied: the bipolar forceps Cardioblate BP (Medtronic) for the radiofrequency, the Epicor Ultracinch LP Ablation device (St. Jude) for ultrasound and the Cardioblate CryoFlex (Medtronic) device for cryoablation. Histological features of lesions made at the pulmonary venous confluence assessed the effectiveness of different energy sources.

**Results:**

Over 45 experimentations performed, only 28 were considered correct and retained for histological analysis. Three distinct groups were studied according to the type of procedure performed: group 1 (Radiofrequency, n = 12), group 2 (ultrasound, n = 4), group 3 (cryoablation, n = 10) and controls (n = 2). All analysed samples showed histological changes with a success rates of transmurality of 33% for radiofrequency, 25% for ultrasound and 90% for cryotherapy (p <0.001). The average length of transmurality, when it was reached and the proportion of transmurality over the total length of the lesion were respectively 12 ± 6 mm and 37 ± 18% for group 1, 10 mm and 33% for group 2 and 11.1 ± 1.1 mm and 35 ± 5% for group 3.

**Conclusion:**

Immediate detectable histological transmural lesions after epicardial procedure are discontinuous whatever the kind of energy source tested in this work and it strongly encourages the repetition of radiofrequency procedures. Nevertheless, our experimental model seems inadequate to assess ultrasound energy efficacy.

## Background

The Cox-maze procedure has proven to be the most effective surgical procedure for treating atrial fibrillation (AF) and its adverse consequences [1,2]. Modifications of the initial Cox-Maze procedure have led to the "cut-and-sew" Cox III method. The work of Haissaguerre [3] demonstrated the effectiveness of radiofrequency ablation in the reproduction of cut-and-sew lines of the Cox procedure. Thus, Cox III has evolved into Cox IV where most of the sutures have been replaced by lesions produced by radiofrequency or other energy sources, in order to decrease operative time, and reduce the haemorrhagic risks associated with atrial sutures [4].

“Mini-Maze” procedures with a success rate at 1 year of 82% [5], simplified the initial plan of Cox, in case of paroxysmal AF and allowed the promotion of epicardial treatment of AF, including beating heart and/or minimally invasive approaches [6,7]. In contrast, permanent AF appears to be maintained by complex reentry circuits defining a new concept of electrical atrial remodelling [8]. Simple pulmonary vein isolation is not sufficient and the complete scheme of Cox appears necessary to treat permanent AF. Three energy sources are commonly used during Cox-Maze IV procedures: radiofrequency, ultrasound and cryoablation gas (Argon). Nevertheless, no consensus exists about the best energy source capable of reproducing transmurality and continuity of cut-and-sew lines [9].

The aim of this experimental work was to evaluate and compare the histologic transmurality of lesions induced immediately by different energy sources from three antiarrhythmic epicardic devices.

## Methods

This study was made possible by the generosity of 45 individuals who donated their bodies to science through CERAN (non-profit association for body donations associated with the anatomy laboratory of Nantes). The donor age ranged between 68 and 82 years (mean = 75.2 ± 2.2 years). 55% (n=25) of subjects were male. The bodies were used "fresh", 48 hours after death (statutory period required). There was no cadaveric solution for preservation of the body.

### Materials

Three anti-arrhythmic epicardial devices corresponding to three different energy sources were evaluated: the Cardioblate® bipolar device (Surgical Ablation System, Medtronic, Inc) for radiofrequency, the UltraCinch LP® Ablation device (Epicor Medical Cardiac Ablation System, Saint Jude) for ultrasound or HIFU (High Intensity Focused Ultrasound), and the Cardioblate CryoFlex® device (Argon-powered Cryoablation, Medtronic) for cryoablation. Each antiarrhythmic device was connected to its specific console: Cardioblate generator (Surgical Ablation System Generator, Medtronic), Epicor Cardiac Ablation System (Ablation Control System (ACS), Saint-Jude) and Cardioblate CryoFlex console (Surgical Ablation Console, Medtronic). The extracorporeal circulation circuit, assembled in a closed circuit (Sarns), was connected to a heat exchanger (Jostra Quadrox) and a heat generator (Stockert), simple back. Myocardial temperature was monitored by a thermal probe, myocardial needle temperature probe (Genesee Biomedical).

### Experimental model

**Figure 1:**
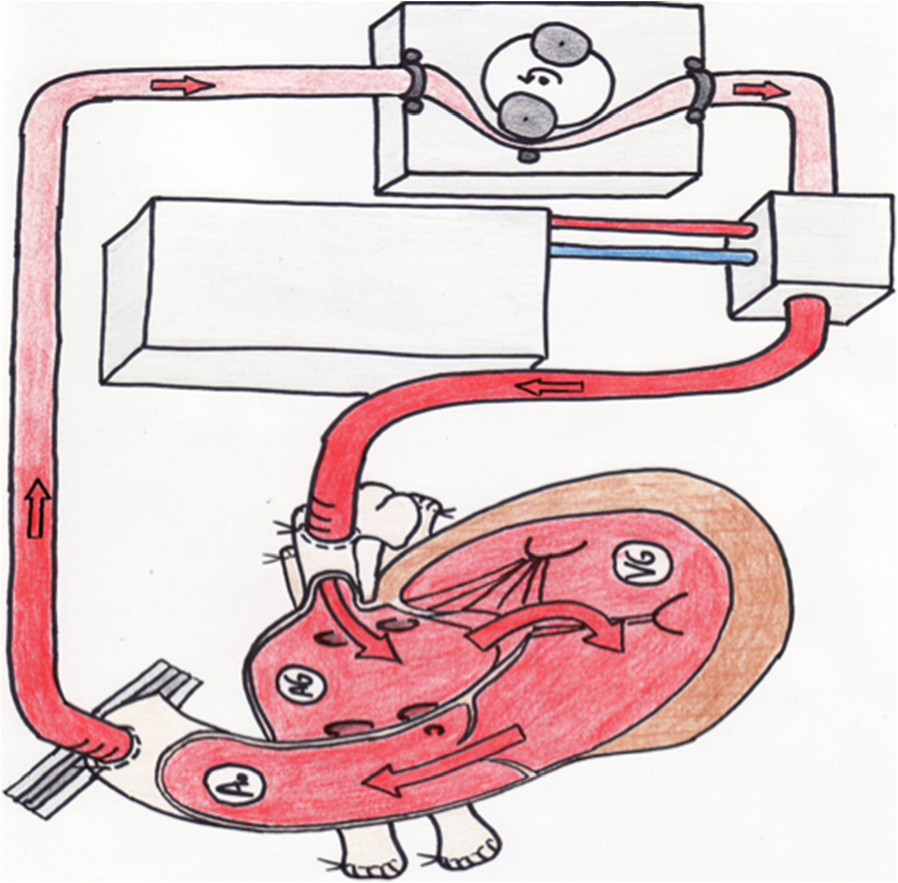
Schematic of the experimental model.

**Figure 2:**
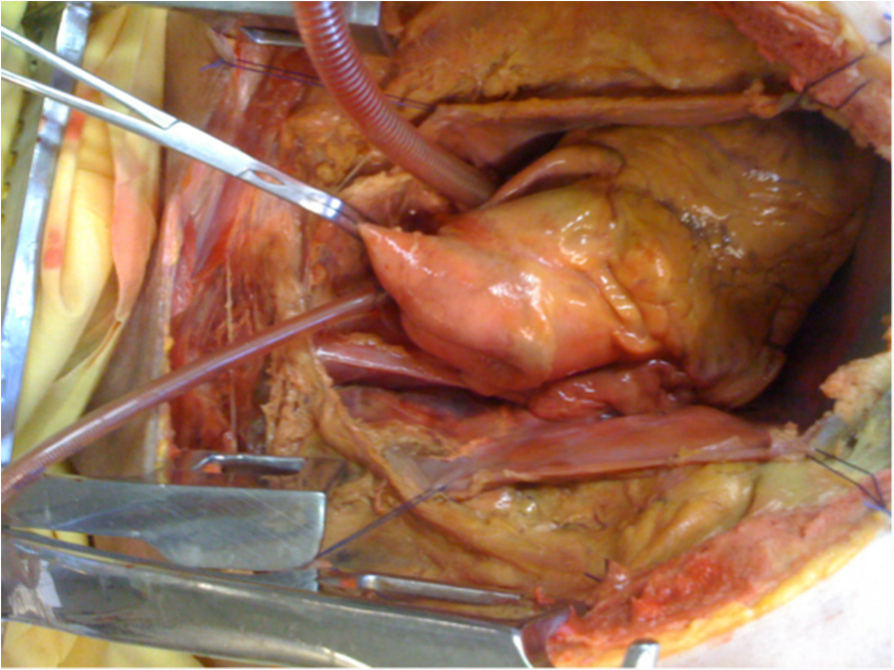
**Aortic clamping and positioning of the cannulas.** The left cavities appear filled unlike the right cavities that are not involved in experimentation.

The objective of this study was to design an experimental model capable of reproducing an epicardial procedure for atrial fibrillation treatment, at 37°C on a cadaveric human heart in the orthotopic position and considered "fresh" (48 hours of ischemic time). The heart cavities were maintained filled through the use of cardiopulmonary bypass. After sternotomy and opening the pericardium, the pulmonary veins were ligated and dissected away from the left atrium. The aorta was dissected and clamped close to the brachiocephalic arterial trunk. An injection cannula, positioned in the left atrium, and a cannula located in the aorta allowed the realization of a closed extracorporeal circulation (Figure 1). The fluid flowing through this closed circuit consisted of 0.9% saline solution. The outflow in the circuit was 3 l/min and allowed the flow in the left cardiac cavities to remain near to physiologic conditions (Figure 2). A coronary circulation could be even observed. This circuit was coupled to a thermal generator through a heat exchange membrane type. The temperature of the assembly was set at 37°C and the myocardial temperature was constantly monitored. Antiarrhythmic procedures were performed when myocardial temperature had reached 37°C.

### Protocol epicardial lesion

The radiofrequency and cryoablation devices took the form of a clamp and the ultrasonic device a collar. The procedure for issuing different energies was modeled on clinical practice. Once the process began, the generator automatically regulated the transmission time for a theoretical transmurality of the lesions. Lesions made with radiofrequency and cryoablation devices showed only a single line on the right and left pulmonary vein confluences. The average time of radiofrequency procedures was 16 ± 1 sec. For cryoablation, epicardial application time of the Cardioblate CryoFlex® device was 60 seconds, as recommended by the former manufacturer. For the ultrasound system, the size of the collar was chosen using the meter supplied in the kit, with the aim of determining the collar that best fitted the morphology of the left atrium. The lesion pattern performed with the ultrasonic collar was the "box lesion", which isolates the bottom of the left atrium and the pulmonary venous confluence from the rest of the left atrium. Once the device was in place, the generator controlled a long process intended to result in the transmurality of the lesion. The average time of application was 10 ± 0.5 min.

### Sampling and histological analyses

The effectiveness of different energy sources was assessed using histologic features of the lesions made with these antiarrhythmic devices. To compare strictly identical tissue samples, two lesion samples were systematically taken from each heart. The first sample extended from the lower edge of the right inferior pulmonary vein to the upper edge of the right superior pulmonary vein at the anterior face of the venous confluence. The second sample was between the lower edge of the left inferior pulmonary vein to the upper edge of the left superior pulmonary vein. Considering that lesions could continue to evolve in the minutes following their induction, histological samples were taken four hours after the creation of lesions. Each lesion sample was associated with a healthy sample of the same tissue. Lesion samples were kept in a solution of formalin 10% for more than 24 hours at 4°C. The inclusion was done in paraffin. Histologic analysis of slides was performed "blind" after classical staining (hematoxylin phloxine saffron [HPS]) without knowledge of the type of energy used, by a pathologist specializing in cardiovascular tissues at the thorax Institute of Nantes University Hospital.

### Criteria for histologic evaluation

**Figure 3:**
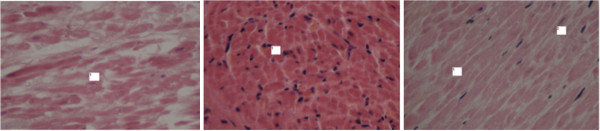
**Magnification x400 HPS staining.** Left: ghost myocytes with no nucleus and blurred cytoplasmic boundaries. Middle: hyperchromatic nuclei. Right: absence of nuclei, viable myocytes.

Transmurality and continuity of the lesions were evaluated histologically. The tissue damage induced by the procedure was defined by the existence of one or more of the following histopathologic aspects (Figure 3): “ghost” myocytes with an absent nucleus or very pale; coagulation necrosis of myocytes with more hyperchromatic eosinophilic with dense nuclei; "patch", involving viable, isolated, and “ghost” or hyperchromatic myocytes. The procedure was defined as transmural only when one or more of these histologic lesions were found throughout the depth of the atrial tissue. The length of tissue with acquired transmurality was assessed by the sum of the transmural lesion areas. The maximum depth of the lesions was also evaluated for each sample. Along with this histologic evaluation, macroscopic esophageal lesions were systematically evaluated after cardiac explantation.

### Creating a control group

In order to confirm that observed histological features on a cadaveric heart were similar to a non-cadaveric heart, a control group was created with different pattern lesions on a beating heart. After approval by the ethics committee of the Nantes University Hospital and patient consent, lesions controls were performed by radiofrequency on a beating heart at the base of the left atrium appendage in a 45 years old patient just before explantation for cardiac transplantation. These lesions *in vivo* were compared with identical lesions done during two additional experimental procedures on perfused cadaveric hearts.

### Exclusion criteria of the study

Procedures that failed to result in a sealed circuit were excluded from the study; these were subjects with aortic stenosis or with intra-cavity thrombus that do not allow proper operation of the circuit. The unsuitability of some atriums to ultrasound clamp sizes resulted in the cancellation of five manipulations.

### Statistical analysis

A non-parametric Kruskal-Wallis test was used. Statistical analysis was performed by MedCalc® software, version 9.3.7.0.

## Results

Of the 45 procedures, only 28 were considered accurate and retained for histologic analysis. Three groups were studied according to the type of energy sources: Group 1 (radiofrequency) consisted of 12 subjects aged between 68 to 82 years (8 men/4 women; mean age 73.6 ± 4 years); Group 2 (ultrasonic) consisted of 4 subjects aged between 75 to 77 years (2 men/2 women; mean age 76±0.8 years) and Group 3 (cryoablation) consisted of 10 subjects aged between 72 to 80 years (6 men / 4 women; mean age 75.8 ± 2.8 years). A control group with left atrial appendage radiofrequency lesions (n=2) was created in order to compare these lesions with those obtained on the heart *in vivo*. There was no significant difference in age between groups (p=0.17). The total length of the histologic samples analyzed by subject and atrial tissue thickness were similar between groups (Table 1). All samples analyzed showed histologic changes, demonstrating the success of the procedure in terms of energy transfer, but depth of lesions depended on the devices. No difference was observed between the three techniques in terms of histologic types of lesions. All tissue samples studied showed the same histopathologic type of myocytes, ghosts or hyperchromatic, portions of patch lesions, loose interstitial tissue and/or pericellular edema (Figure 3).

**Table 1:** Average of total lengths of histologic samples analyzed per group and mean thicknesses of atrial tissue

	**Group 1 (n=12)**	**Group 2 (n=4)**	**Group 3 (n=10)**
**Total length of the histological samples analyzed by subject**	**33.7 ± 3.3 mm**	**33.7 ± 2.5 mm**	**33.1 ± 3.4 mm**
**Thickness of atrial tissue**	**3.71 ± 1.0 mm**	**3 ± 0.7 mm**	**3.1 ± 0.46 mm**

**Figure 4:**
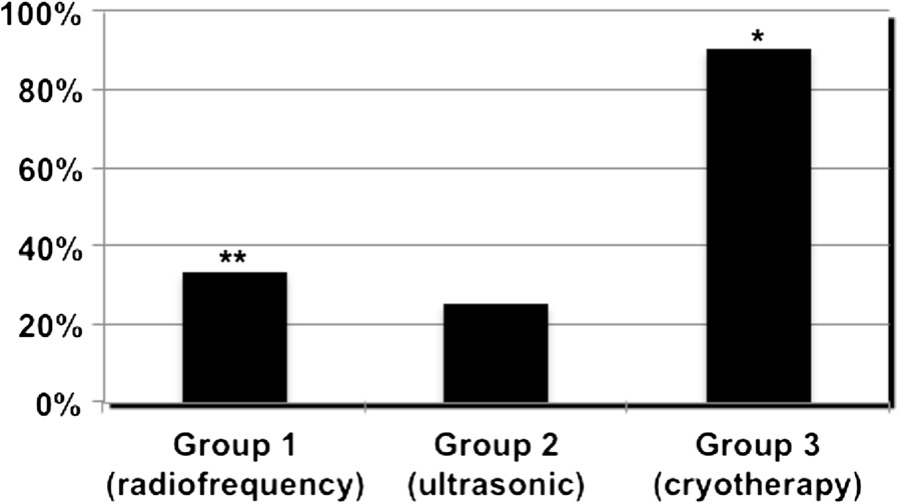
Percentage of procedures in which transmurality histological lesions were found according to the type of energy used (* significantly difference between cryotherapy against both radiofrequency and ultrasound (p=0.0053); ** no statistical difference between radiofrequency against both cryotherapy and ulstrasound (p=0.11)).

The percentage of procedures in which transmurality was found in histologic lesions is shown in Figure 4. In group 1, immediate histologic transmurality was found in 33% (n=4) of the procedures with radiofrequency. Among these four samples with transmurality lesions, gaps in the radiofrequency line were always observed and the average length of lesions with transmurality was 12 ± 6 mm per sample, representing 36.5% of the ablated atrial tissue length. Among samples with no transmural lesion (n=8), the mean maximum depth of lesions observed was 0.68 ± 0.3 mm for an average tissue thickness of 3.8±1 mm, representing a depth penetration of 19±11%. In group 2, only 1 (25%) out of 4 procedures had transmural lesions with a length of 10 mm representing 33% of the atrial tissue length. Among the 3 procedures with no transmural lesion, the mean maximum depth of lesions was 0.41±0.3 mm for an average thickness of 2.6±0.2 mm, representing a depth penetration of 14.8±10%.

At last, 90% of samples (n=9) in the group 3 had transmural lesions, with an average length of transmural lesions of 11.1±1.1 mm, representing a transmurality length of 35±5% of the atrial tissue.

The macroscopic analysis of the heart and mediastinum found no esophageal necrosis or perforation after the atrial antiarrhythmic procedure.

To validate this model and histologic analysis of lesions, a radiofrequency lesion was performed on the basis of the left atrium in a control patient awaiting heart transplantation and compared with two isolated radiofrequency lesions of the left atrium of two experimental subjects. Histopathologically, the lesions observed on the left atrium of the control patient were similar to lesions observed in the experimental model, but showed no transmural aspect. The bulk of the lesion areas were made of patch strips while the two left atrial samples from the experimental model showed transmural lesions (Table 2).

**Table 2:** Results of the left atrial control groups

	**Lesion length with transmurality (mm)**	**Length of samples without transmurality (mm)**	**Thickness of tissue (mm)**
**Patient 1**	**18**	**20**	**3**
**Patient 2**	**16**	**14**	**2,5**
**Patient awaiting transplant**	**0**	**30**	**4,5**

## Discussion

Several experimental models on the effectiveness of different histologic energy sources have already been made, either in animal models or in conditions not comparable to that of a heart in “charge” [10,11]. Our experimental model has the advantage of reproducing the conditions of a heart “in charge” at 37°C. There is therefore no protective cooling effect as suggested by some experimental work showing that tissue temperatures below 37°C decreased the transmurality of the lesions when using hot energy such as radiofrequency and ultrasound [12]. However, none of the epicardial devices evaluated in our experimental model causes an immediate and continuous transmural lesion on a whole sample. The histologic interpretation in acute and early phase certainly underestimates the importance of the lesion [13], and the electrophysiologic consequences of scarring induced late after the procedure. Otherwise, the clinical efficacy of transmurality was also questioned by some clinical and experimental studies [12,14-18]. Several hypotheses were raised: new concepts of electro-anatomic remodeling of the atria associated with the use of these energy sources; no justification of transmurality of lesions according to the endocardial or epicardial site of delivery of energy or the type of AF (permanent or paroxysmal) [19]? One could indeed imagine that the electrical isolation of arrhythmia triggers in the pulmonary veins does not necessarily justify the transmural lesions in cases of paroxysmal AF. The fact remains that it is the lesion induced by the energy source that will determine progress of the wave of depolarization through the atrium and thus the effectiveness of the Maze and the electro-anatomic remodeling. This discontinuity of transmurality has already been demonstrated for microwaves [17] and may partly explain the occurrence of AF recurrence for other energy sources.

In addition, transmurality of the lesions significantly depends on the type of energy used in our model, with immediate transmurality success rates of 25–30% for radiofrequency and ultrasound against 90% for cryoablation (p<0.001).

Cryoablation could be even more efficient since the new recommended application time has increased from 60 sec to 2 minutes. The success rates of immediate transmurality are small for both radiofrequency and ultrasound, whereas in clinical practice, the studies did not find any clear superiority of cryoablation compared with other energies in terms of recurrence of AF [20-22].

In fact, the histologic findings of our experimental model corroborate experimental work that demonstrated a difference in intensity of the lesions induced by different types of energy. Rodriguez *et al.* showed in an animal model of atrioventricular node ablation that cryoablation induced histologic lesions that were more homogeneous and more intense than those induced by radiofrequency [23]. However, the initial importance of histologic lesions does not condition electrophysiologic long-term success [24,25] and some studies have even observed poorer outcomes with cryoablation versus radiofrequency in the long term [24,25]. These results should be interpreted with caution. They are obtained thanks to nitrous oxide which induces lesions at −60°C. Epicardial cryoablation is based on Argon and cooled to −160°C [19]. The interpretation of our results obtained with radiofrequency could also call into question the validity and effectiveness of our model. We therefore examined the transmurality of radiofrequency-induced lesions of the left atrium on a beating heart (given the impossibility to collect at the pulmonary venous confluence), just before cardiac explantation. No histologic transmural lesion could be found in this case while two experimental procedures controls were allowed to find a transmurality of lesions at the base of the left atrium. Variability in the amount of adipose tissue may partly explain these results. The thickness of adipose tissue could reduce atrial transmurality of radiofrequency lesions [12].

Finally, even if our results with RF does not match the animal experimental results (99% of the lesions were transmural in pig heart [26] and 92% of the lesions were transmural in ovine heart [16]), they seem to corroborate the human electrophysiological results of Benussi [27]. He advises to repeat the procedure 3 times around the pulmonary veins before getting a block, witness of transmurality.

On the other hand, the rather unfavourable results obtained with Epicor, question the validity of our experimental model for the assessment of ultrasound. "Non-conductive" energy, based on the action of HIFU, causes molecular agitation, nuclear and cell destruction. It is designed to operate on a beating heart, not on ischemic hearts or non-beating heart. The discrepancy between our results and important animal experimental work [28] or human histological post-mortem work [29] seems to invalidate our experimental protocol for the study of ultrasound.

Effectiveness of epicardial lesions, whatever the type of energy used, could also explain the results, but a comparative study, endocardial vs. epicardial energy delivery, should be performed to confirm this hypothesis.

Finally, we have noticed no associated mediastinal lesions, particularly in the oesophagus. An epicardial procedure, with the orientation of the energy source to the atrial cavity (unlike the way in which the endocardial source of energy is directed into the oesophagus), seems to confirm the benign nature of mediastinal structures.

## Conclusion

Despite the apparent superiority of cryoablation over other energy sources, the immediate histologic transmural lesions obtained in our experimental model remain generally limited or discontinued after an epicardial procedure. Our results clearly encourage the repetition of the radiofrequency procedure to obtain a conduction block. These findings support also the development of hybrid procedure to complete discontinued lines. Nevertheless, our model seems inappropriate to the study of ultrasound.

## Abbreviations

AF: Atrial fibrillation; HIFU: High intensity focused ultrasound; HPS: Hematoxylin phloxine saffron.

## Competing interests

The authors declare that they have no competing interests.

## Authors’ contributions

JMEA designed and performed the study. CT performed histological analysis. JCR, OB, TS, AM and OH were involved in the interpretation of the data and revised the article critically. All the authors gave their final approval of the version to be published.
